# Comprehensive meta-analysis of Signal Transducers and Activators of Transcription (STAT) genomic binding patterns discerns cell-specific *cis*-regulatory modules

**DOI:** 10.1186/1471-2164-14-4

**Published:** 2013-01-16

**Authors:** Keunsoo Kang, Gertraud W Robinson, Lothar Hennighausen

**Affiliations:** 1 Laboratory of Genetics and Physiology, National Institute of Diabetes and Digestive and Kidney Diseases, National Institutes of Health, 8 Center Drive, Bethesda, MD, 20892-0822, USA; 2 National Department of Nanobiomedical Science and WCU Research Center of Nanobiomedical Science, Dankook University, Cheonan, Chungnam, 330-714, Republic of Korea

**Keywords:** STAT, GAS motif, Meta-analysis, ChIP-seq, *Cis*-regulatory module, CRM

## Abstract

**Background:**

Cytokine-activated transcription factors from the STAT (Signal Transducers and Activators of Transcription) family control common and context-specific genetic programs. It is not clear to what extent cell-specific features determine the binding capacity of seven STAT members and to what degree they share genetic targets. Molecular insight into the biology of STATs was gained from a meta-analysis of 29 available ChIP-seq data sets covering genome-wide occupancy of STATs 1, 3, 4, 5A, 5B and 6 in several cell types.

**Results:**

We determined that the genomic binding capacity of STATs is primarily defined by the cell type and to a lesser extent by individual family members. For example, the overlap of shared binding sites between STATs 3 and 5 in T cells is greater than that between STAT5 in T cells and non-T cells. Even for the top 1,000 highly enriched STAT binding sites, ~15% of STAT5 binding sites in mouse female liver are shared by other STATs in different cell types while in T cells ~90% of STAT5 binding sites are co-occupied by STAT3, STAT4 and STAT6. In addition, we identified 116 *cis*-regulatory modules (CRM), which are recognized by all STAT members across cell types defining a common JAK-STAT signature. Lastly, in liver STAT5 binding significantly coincides with binding of the cell-specific transcription factors HNF4A, FOXA1 and FOXA2 and is associated with cell-type specific gene transcription.

**Conclusions:**

Our results suggest that genomic binding of STATs is primarily determined by the cell type and further specificity is achieved in part by juxtaposed binding of cell-specific transcription factors.

## Background

In Drosophila the single STAT (Stat92E), in conjunction with one cytokine (UPD), controls an array of developmental processes ranging from immune responses and heart development to the specification of border cells in the ovary and primordial germ cell formation in the gonads [1]. In contrast, mammals have seven STATs (1–4, 5A, 5B and 6) [2]. Although these STATs recognize similar, if not identical, DNA sequence motifs *in vitro* they execute cell- and context-specific functions in addition to overlapping and redundant functions. Yet, cell-specific gene expression patterns are obtained despite different cells being exposed *in vivo* to similar, and in some cases identical, cytokines. The appropriate execution of these programs is determined by several regulatory layers [3]. These include a large number of membrane receptors that have the ability to differentially activate individual STATs, cellular STAT levels, the affinity of STATs to receptors and their cognate JAKs and possibly the ability of STATs to recognize regulatory sequences only in certain contexts, such as composite promoter elements or chromatin configuration. In fact, evidence is emerging that specific chromatin remodeling is required for STAT binding to a subset of loci [4,5].

Direct STAT binding to cognate genomic targets will, at least in part, execute cytokine stimuli. With this in mind, new and critical insight into common and cell-specific functions of STATs could come from genome-wide STAT occupancy data sets. However, it is not clear to what extent different members of the STAT family share genetic targets. In particular STAT binding to the canonical GAS (gamma interferon-activated sequence) motif (TTCnnnGAA), the extent of cell specificity and the influence of STAT concentration on their ability to occupy genomic sites are poorly understood. Large-scale chromatin immunoprecipitation followed by high throughput sequencing (ChIP-seq) studies have explored *in vivo* binding of five different STATs in a number of different cell types exposed to several cytokines. We have now comparatively reanalyzed this resource of 29 data sets and provide insight into the complexity of common and selective STAT binding patterns that are unique to, as well as shared between, different cell lineages.

## Results and discussion

### Meta-analysis of ChIP-seq data sets reveals cell context as the major defining factor controlling STAT binding to specific GAS sites

The Signal Transducer and Activator of Transcription (STAT) family consist of seven transcription factors (TFs) called STAT1, STAT2, STAT3, STAT4, STAT5A, STAT5B and STAT6, which upon activation by cytokines bind to specific sequences called GAS motifs (TTCnnnGAA) [3,6,7]. To determine the extent of genomic binding of each STAT member in various cell contexts, we collected available STAT (1, 3, 4, 5 and 6) ChIP-seq and control data sets from 11 independent studies (gene expression omnibus, http://www.ncbi.nlm.nih.gov/geo/) [8-19] and re-analyzed them using the same analysis pipeline (Additional files 1, 2 and Methods). Since the number of significant peaks is sensitive to algorithms [20-22], we used three different peak-calling programs, MACS (version 1.4.2), HOMER (version 3.10) and Qeseq (version 0.2.2) as Chen et al. suggested [20,21,23,24]. The combined peaks were categorized into three classes (high-, intermediate- and low-confidence) according to the number of algorithms that detected the peaks (Additional file 1). In this regard, the high- and intermediate-confidence peaks should be more reliable than low-confidence peaks due to the fact that any two different algorithms identified them as significant peaks (good signal-to-noise ratio).

**Figure 1:**
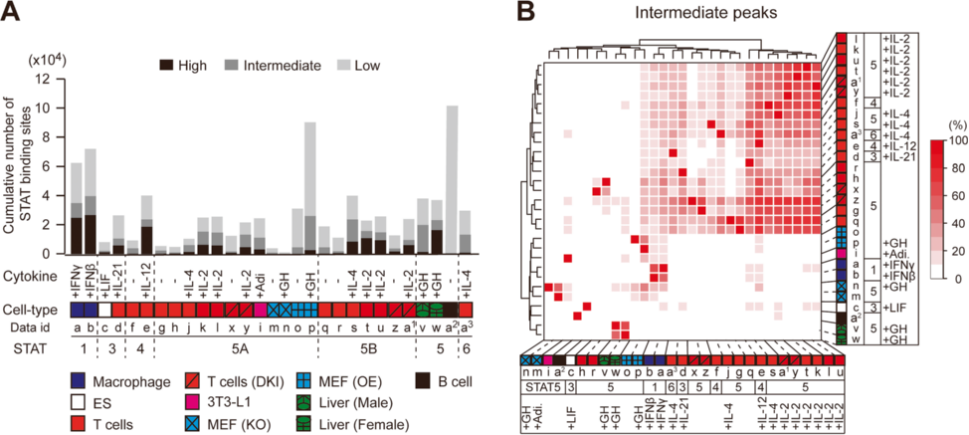
**Genome-wide STAT binding sites in different cell-types.** (**A**) The number of STAT binding sites identified in various cell types is shown as a bar graph ranging from few hundreds to one hundred thousand. STAT binding sites in each sample were categorized into three confidence classes (high, intermediate and low) based on the number of detections by three peak-calling algorithms, MACS, HOMER and Qeseq. Detailed information (data ids) about ChIP-seq data sets used in this study can be found in Additional file 2, and the analysis pipeline is described in Additional file 1. (**B**) Heat map shows the unsupervised clustering of 29 samples according to genome-wide STAT binding sites, using a 500-bp window. The percentage of overlapping sites between two samples (x-axis over y-axis) was calculated and used to draw the heat map. OE, Stat5^−/−^ MEFs overexpressing STAT5A; DKI, STAT5 mutant that prevents the formation of tetramers; KO, STAT5A knock-out; MEF, mouse embryonic fibroblast; ES, embryonic stem cells; 3T3-L1, pre-adipocyte cells; GH, growth hormone; IL, interleukin; Adi., adipogenic inducers; IFNγ, interferon-gamma; IFNβ, interferon-beta; LIF, Leukemia inhibitory factor.

Comparative analysis of genome-wide STAT binding data sets validated the cytokine-dependent nature of STAT binding to DNA. STAT activation by cytokines induced a large number of genomic binding sites compared with corresponding unstimulated controls in all cases, regardless of the cell type and cytokine with the exception of STAT5A-null cells (Figure 1A). The treatment of STAT5-null cells with growth hormone yielded no evident changes in the number of STAT5 binding sites. Even for the same STAT, the number of binding sites greatly varied between different cell contexts. The total number of STAT enriched binding sites ranged from several hundred to one hundred thousand depending on the cell type (Additional file 2). As expected, low-confidence peaks (a collection of peaks identified by any algorithm) seem to be unreliable in some data sets (MEFs and B cells). Therefore, we only used high- and intermediate-peaks for the rest of the analyses. In addition to the quantitative aspect of genome-wide STAT binding, we determined that cell context was the foremost defining factor in the establishment of genome-wide binding positions of STATs. To estimate overall similarity of global STAT binding sites, we performed unsupervised hierarchical clustering of 29 cell contexts based only on their genome-wide binding sites (Figure 1B). These two-way comparisons elucidated the extent of distinct and overlapping STAT occupancy between different cell types and individual STATs. For instance, up to 18 samples obtained from T cell lineages with different STAT members (3, 4, 5 and 6) and/or cytokine treatments (IL-2, IL-4, IL-12 and IL-21) constituted the largest cluster on the heat map, whereas other cell contexts including MEFs (mouse embryonic fibroblasts) and ES (embryonic stem) cells were distinct from this T cell group. This result demonstrates that the genomic binding capacity of STATs is primarily defined by the cell type and less so by the individual STAT protein. For example, the overlap of common binding sites between STATs 3 and 5 in T cells (up to 43%) is greater that between STAT5 in T cells and non-T cells (up to 17%).

### STATs regulate gene expression by cell-specific binding to distinct sets of GAS motifs

**Figure 2:**
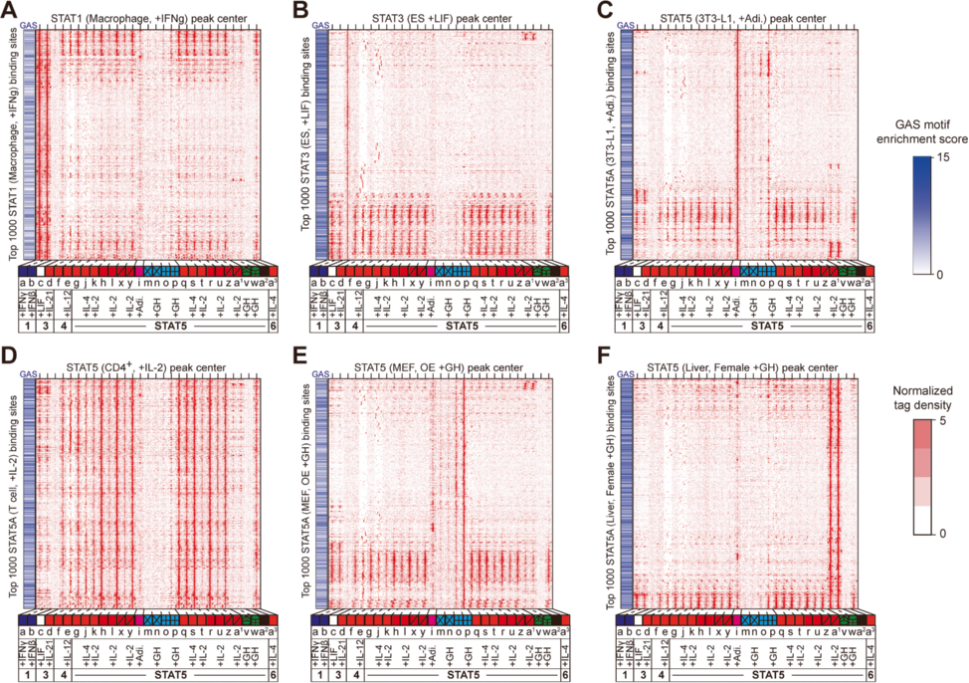
**Shared and distinct sites of occupation.** Hierarchical clustering (average linkage algorithm) was performed with normalized ChIP-seq tags (tags per 10 million), which are located within +/− 1.5 kb flanking regions of the top 1000 STAT peak centers (determined by height of peaks in each condition). GAS motifs were identified within +/− 75 bp flanking regions of the top sites using the MOODS algorithm (p value < 0.01) [26]. Rows represent the top 1000 STAT binding sites in the given cell type (top label) and red vertical lines in tracks indicate the binding of STAT under specific conditions (bottom labels). (**A**) STAT1 peak centered in macrophages with IFNγ stimulation. (**B**) STAT3 peak centered in ES cells with LIF stimulation. (**C**) STAT5 peak centered in 3T3-L1 cells with adipogenic inducers. (**D**) STAT5 peak centered in T cells with IL-2 stimulation. (**E**) STAT5 peak centered in STAT5A overexpressing MEF with growth hormone. (**F**) STAT5 peak centered in female liver with growth hormone.

The extent to which different members of transcription factor families bind to and occupy identical sites within the genome is a central issue, as different members of a given family activate distinct and cell-specific genetic programs [25]. In order to assess the extent of cell-type specific occupation of GAS sites and infer their roles in gene regulation, we first identified all GAS motifs around the peak center of STAT binding sites (+/− 75 bp) using the MOODS algorithm [26] with the known STAT position frequency matrix (p value < 0.01, JASPAR matrix id; MA014431 from http://jaspar.cgb.ki.se/) [27]. Then, we measured ChIPed-tag density of all 29 ChIP-seqs around the center of the top 1,000 highest peaks (+/− 1.5 kb flanking regions) in six representatives of the different cell types. This analysis demonstrated the existence of shared and cell-type specific STAT binding sites with GAS motifs (Figure 2). For each cell type approximately 50-85% of the STAT binding sites were unique, even though most (more than 88%) of the sites contained GAS motifs within a 75 bp perimeter from the center (Figure 2). For instance, 65% of STAT3 binding sites in ES cells did not coincide with the binding of any of the other STATs in 28 different contexts (Figure 2B), while STAT binding sites in T cells largely overlapped (Figure 2D). In female liver, less than 15% of STAT5 binding sites were shared with any other STATs in different cell types (Figure 2F). This cell-type specificity of STAT binding may contribute to the regulation of cell-type specific genes, which are involved in differentiation or developmental processes. To address this question, we assessed statistically significant functions of genes within flanking regions of STAT binding sites in six different cell types using the GREAT program [28]. Each set of STAT binding sites was indeed located near the genes that are particularly important for the respective cell types (Additional file 3). In ES cells STAT3 bound to GAS sites near genes involved in differentiation, maintenance and development of stem cells (binomial Bonferroni *P-value* < 1.9 × 10^-5^) and genes that are expressed in very early embryonic stages (Additional file 3). In female liver, STAT5 binding sites coincided with GAS motifs located near genes contributing to metabolic processes, such as organic acids, carboxylic acids and lipid metabolic pathways (binomial Bonferroni *P-value* < 2.4 × 10^-7^). Notably, the majority of these genes were specifically expressed in liver (Additional file 3). Overall, cell-type specific recognition of GAS sites appears to be essential for maintaining cell identity as well as promoting proliferation and differentiation in response to cytokine signals.

### Gene sets targeted by all STAT members independent of cell type and cytokine stimulus generate a JAK-STAT signature

**Figure 3:**
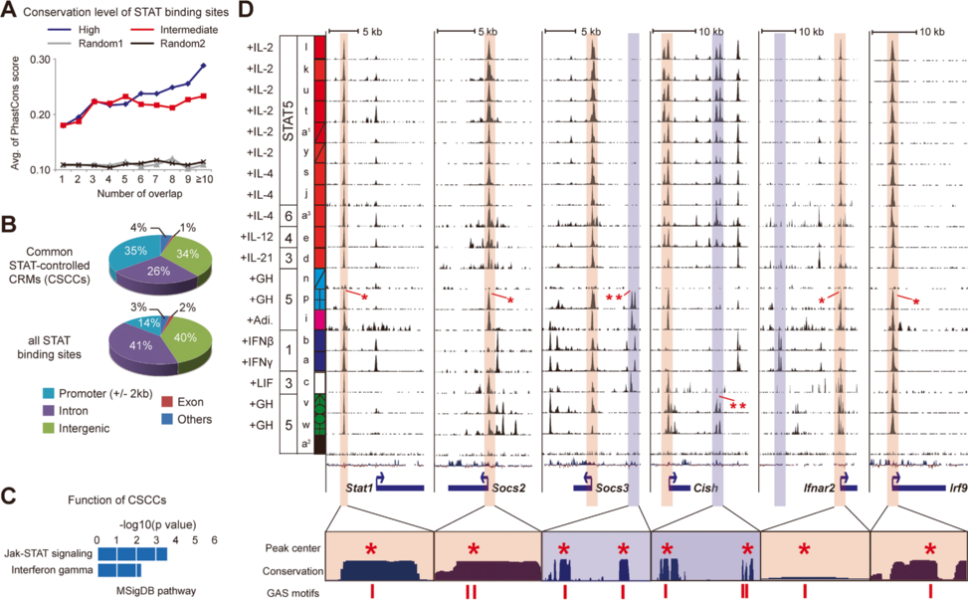
**Characterization of common STAT binding sites.** (**A**) Sequence conservation of shared STAT binding regions was calculated and averaged using PhastCons score. The x-axis indicates the number of overlaps by any of the STAT binding sites. Two random sets were generated (same number of regions – high / intermediate sets) for comparison. Pearson’s correlation coefficient r = 0.95 (high), r = 0.69 (intermediate), r = 0.04 (random1) and r = 0.53 (random2). (**B**) Distributions of STAT-associated CRMs and all STAT binding sites were estimated. The ‘others’ category includes 5^′^- and 3^′^-UTRs. (**C**) Statistically significant pathways associated with the genes near CRCCs were inferred using the GREAT tool with the following default settings; Basal plus extension – proximal: 5 kb upstream and 1kb downstream / distal: up to 1000 kb [28]. P-value is the Bonferroni corrected binomial P-value. (**D**) STATs 1, 3, 4, 5 and 6 can bind to the same sites in similar or different cell contexts. Six loci encoding *Stat1*, *Socs2*, *Socs3*, *Cish, Ifnar2* and *Irf9* are shown. Red vertical bars overlapping peaks indicate the binding regions of STATs 1, 3, 4, 5 and 6 shared between several cell types, while blue vertical bars represent the sites with unique context-dependent STAT binding. The bottom panel depicts positions of peak summits and sequence conservation as well as GAS motifs (TTCnnnGAA, perfect match) at the STAT binding regions. The total number of tags in ChIPed samples was normalized to the corresponding input using the wignorm program [24] and the y-axis scale was adjusted according to this normalized value using auto-scale function of the UCSC genome browser. Red asterisks denote peak centers.

The high degree of coinciding genomic occupancy by several, if not all, STAT members in different T cell populations suggests that distinct cytokines are likely to control similar and overlapping gene sets. To characterize these gene sets, all STAT binding sites from the 29 ChIP-seq data sets were integrated to define genome-wide c*is*-regulatory modules (CRMs) associated with STAT members. In general, CRMs are DNA fragments that are recognized by more than one transcription factor [29]. Therefore, we also asked whether additional transcription factors co-occupy these CRMs (shown later). The degree of evolutionary conservation of these CRMs between species measured by PhastCons score [30] was positively correlated with the number of overlaps (Figure 3A). In general, high-confidence CRMs (STAT binding sites) were more conserved than intermediate-confidence sites (Figure 3A). Given the extent to which different STAT members bound to identical genomic sites, the most conserved of these CRMs were of particular interest since they might constitute key *cis*-regulatory modules targeted primarily by any of the STATs. To elucidate these CRMs called common STAT-controlled CRMs (CSCC), we identified genomic regions recognized by STATs from at least 20 different ChIP-seq data sets. A total of 116 CSCCs were identified and 169 genes are located around the CSCCs (Additional file 4). 41 out of 169 genes were located near the CSCCs which were simultaneously shared by STATs across six representative cell types as shown in Figure 2. The majority of 116 CSCCs were located in promoter (−2 kb ~ TSS ~ +2 kb), intergenic and intronic sequences (Figure 3B). The CSCCs tend to be located within promoter regions as compared with the distribution of all STAT binding sites. Functional clustering of nearby genes illustrated that these CSCCs were significantly associated with JAK-STAT signaling and interferon-gamma signaling pathways (binomial Bonferroni *P-value* < 6.2 × 10^-3^) (Figure 3C). Specifically, the *Stat1*, *Socs2*, *Socs3*, *Cish* and *Irf9* genes, which are bona fide components of the JAK-STAT signaling pathway [31], harbored these CSCCs in promoter proximal regions (Figure 3D). All CSCCs were part of highly conserved sequences in vertebrates, and the peak centers of STAT binding and GAS sites coincided. Although these CSCCs were recognized by the majority of STATs in different cell contexts, we also detected cell-type specific CRMs which seem to be recognized by STATs in only a few cell types. For example, two highly conserved CRMs in the *Socs3* upstream region were recognized only by STAT5 in MEFs and 3T3-L1 cells, by STAT1 in macrophages and by STAT3 in ES cells. On the other hand, STAT4/5/6 in T cells and STAT5 in liver tissues specifically bound to sites in the *Cish* downstream region (Figure 3D). These common and cell-specific bindings of STATs to GAS sites in CRMs likely reflect context-dependent gene regulations in different cell types via STATs.

### Distinct transcription factors work in concert with STATs

**Figure 4:**
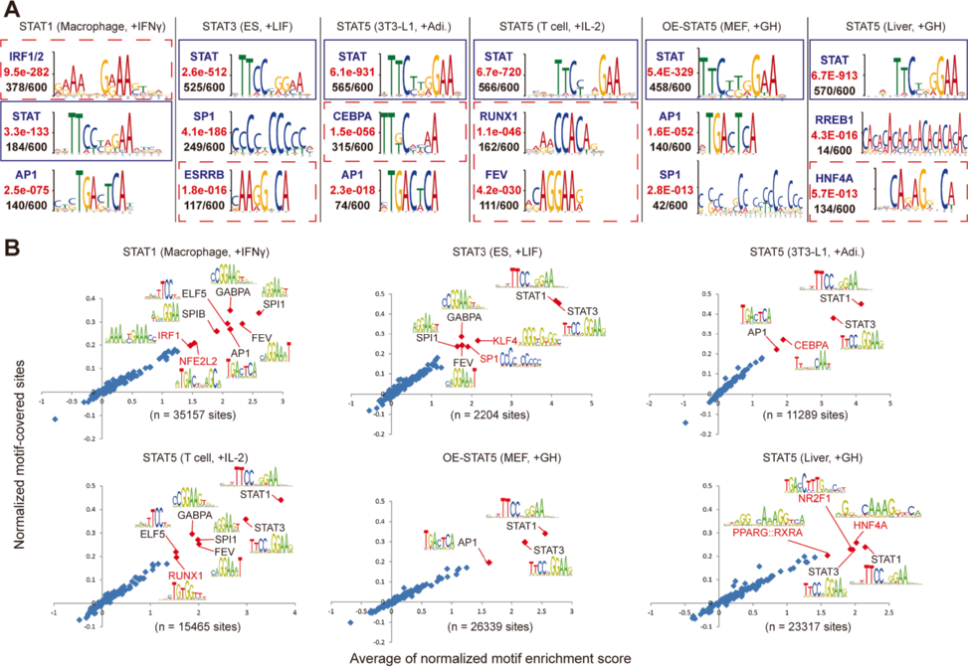
**Cell specificity of transcription factor binding motifs associated with STATs.** (**A**) Distinct sets of specific TFBSs were identified in six representative cell types (Figure 2) within +/− 75 bp of the STAT binding peak centers using the MEME-ChIP *de novo* motif identification program [35]. Top three motifs are shown. The statistical significance of the motifs was estimated using *E-value* (red) and motif occurrence (black) as described previously [35]. The blue and red dashed boxes indicate the identified GAS motifs and cell-type specific TF binding motifs, respectively. (**B**) All motif occurrences on the intermediate-confidence STAT binding sites (+/− 75 bp of the peak center) were calculated using the MOODS algorithm with 130 position-frequency matrices available on the JASPAR website (p value <  0.001). The x-axis and y-axis indicate the mean average of normalized motif enrichment scores and motif-covered sites, respectively. Each dot represents a single TFBS and red dots show significantly associated co-TFs (normalized proportion > 0.2 and average of normalized motif enrichment score > 1.5). Red letters indicate TFBSs unique to the given cell-type.

Recent studies have revealed that several transcription factors (TFs) bind to the same *cis*-regulatory modules, thereby regulating nearby genes [32,33]. Although the seven STAT members recognize the same nucleotide consensus motif of TTCnnnGAA, except for STAT6 (TTCnnnnGAA) [34], distinct sets of TFs might co-localize with STATs and contribute to the diversity of STAT binding sites depending on cell context. To address this question, we first identified the top three significantly over-represented motifs within +/− 75 bp flanking regions of the center of the top 600 STAT binding sites (ranked by peak height from MACS) in six representative cell-types (Figure 2) using the MEME-ChIP program (Figure 4A) [35]. This *de novo* motif identification analysis successfully identified the GAS motif in all cell contexts as significant (Figure 4A, blue box). In addition, unique sets of known TFs verified by the TOMTOM algorithm [36] were detected along with GAS motifs (Figure 4A, red dashed box). The same analysis with STAT4 and STAT6 ChIP-seq sets also identified GAS motifs as the most over-represented motifs validating our motif analysis scheme (Additional file 5). Upon IFNγ induction, the IRF1/2 (interferon regulatory factor 1 or 2) binding motif was the most significant motif associated with STAT binding sites in macrophages but not in other cell types, while the ESRRB (estrogen-related receptor beta) binding motif was only seen in ES cells upon LIF (leukemia inhibitory factor) treatment. Binding motifs for RUNX1 (Runt-related transcription factor 1), which is expressed in T cell lineages [37], were identified in T cells upon IL-2 induction. Many STAT5-bound sequences (134 out of 600) in growth hormone stimulated liver contained HNF4A (hepatic nuclear factor 4 alpha) binding motifs that were frequently found near liver specific genes [38]. These results are consistent with previous reports [10,11,16,39,40]. Additionally, investigation of all the STAT binding sites in different cell types demonstrated that these particular combinations of TFs were not only limited to subsets of the sequences where a given STAT was highly enriched (top 600 sequences) but also could be found in the majority of STAT5 binding sites in specific cell types (Figure 4B).

**Figure 5:**
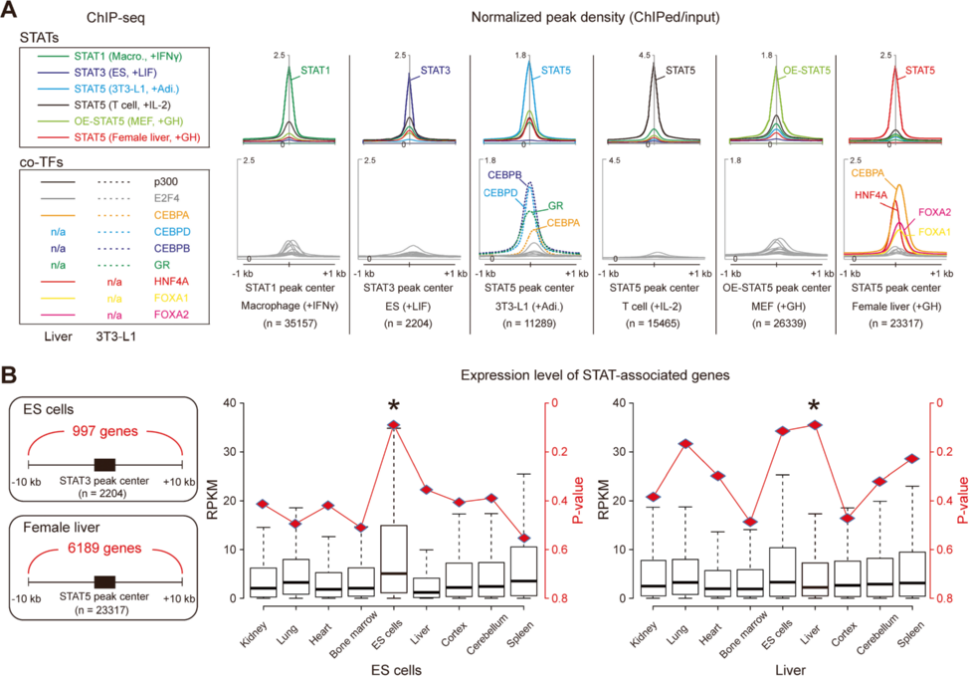
**Cell-specific combinations of STAT5 binding with other transcription factors in mouse liver and 3T3-L1 cells.** (**A**) Average fold change graphs of nine TF ChIP-seq data sets were superimposed on STAT binding sites of six representative cell-types (Figure 2). The fold change was calculated using the wignorm program which estimates fold change between treatment (ChIPed) value to local bias (control, either IgG or input DNA) [24]. Non-significant graphs were colored grey (bottom). The following data sets were downloaded from the GEO website and processed; GSE17067 – p300, E2F4, CEBPA, FOXA1 and FOXA2 in liver and p300, E2F4 and CEBPA in 3 T3-L1 cells; GSE22078 – HNF4A in liver; GSE27826 – CEBPB, CEBPD and GR in 3 T3-L1 cells. (**B**) Expression level (RPKM, reads per kilobase of transcript per million mapped reads) of genes in each tissue was measured using the Cufflinks program [45] with an available RNA-seq data set (GSE29278) [46]. The number of genes located near given STAT-associated CRMs (−10 kb ~ TSS ~ +10 kb) is shown (left panel). These genes were defined as STAT-associated. P-value was empirically calculated by the Monte Carlo simulation [47] with 10000 iterations. The same number of tested genes was randomly selected. Bar graph and red rhombus represent expression levels of STAT-associated genes and the P-values in the given cell type, respectively. Asterisk, P-value < 0.1.

In order to establish whether these CRMs are recognized by TFs *in vivo*, we integrated nine TF ChIP-seq experiments from mouse liver and 3T3-L1 cells [41,42] and drew peak density graphs over the STAT binding sites of six representative cell types (Figure 5A). As predicted by our analysis, CEBP (CCAAT/enhancer-binding protein) A and HNF4A highly occupied STAT5 binding sites in 3T3-L1 cells and liver, respectively. Additionally, binding of CEBPB, CEBPD and GR (glucocorticoid receptor), which are key TFs during early adipogenesis [15], co-localized with STAT5 in 3T3-L1 cells. Also, binding of CEBPA, FOXA1 (forkhead box protein A1) and FOXA2, key regulators in initiating liver specification [43,44], coincided with STAT5. However, E2F4 and p300 (E1A binding protein p300) were not related to STAT5 binding in any cell type.

**Figure 6:**
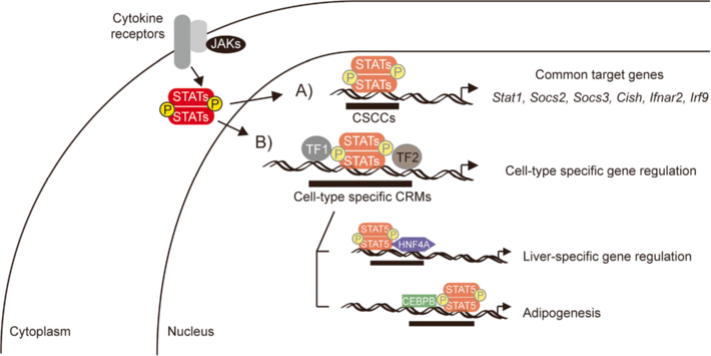
**Model defining STAT-mediated common and cell-type specific gene regulation.** STATs regulate gene sets upon binding to cognate GAS sites located in STAT-controlled CRMs (SCCs). **A**) Common Global STAT targets. 116 common STAT controlled CRMs (CSCCs) have been identified. These CSCCs bind any STAT member in every cell type tested. **B**) STAT controlled cell-specific CRMs. STAT binding coincides with cell-specific transcription factor binding as exemplified for liver and adipocytes. STAT5 binding in liver tissue and adipocytes coincides with genes that are also recognized by HNF4A and CEBPB, respectively.

To validate whether these CRMs are correlated with expression of cell-specific genes, we incorporated nine RNA-seq data sets derived from various tissues [46]. The significance of an association between the CRMs and gene expression was assessed by calculating empirical P-values from Monte Carlo procedures with 10000 iterations for each tissue as described previously (Figure 5B and Methods) [47]. We found that these CRMs were significantly enriched near genes with higher expression levels in the respective cell type. For instance, 997 genes near STAT3-associated CRMs in ES cells were highly expressed in the ES cells (P-value = 0.08) but not in the other cell types, whereas expression of 6189 genes near STAT5-associated CRMs in liver was significantly elevated in liver (P-value = 0.08). These results highlight that defined sets of cell-type specific transcription factors and STATs cooperate via cell-type specific *cis*-regulatory modules to generate cell specific gene expression pattern, whereas STATs control the JAK-STAT signaling related genes via the CSCCs (Figure 6).

## Conclusions

In this study, we explored the extent to which GAS motifs throughout the mammalian genome are occupied by any given STAT in various cell types subjected to different cytokine stimuli. Due to a paucity of available STAT1, STAT4 and STAT6 ChIP-seq data, results were mainly derived from STAT5 ChIP-seq data sets. Our meta-analysis confirmed the increment of STAT binding on GAS sites upon cytokine induction (Figure 1A). Most of the up to 100,000 sequences occupied by STATs (up to 94%) contained a GAS motif (Additional file 2). Since the mouse genome harbors more than one million GAS sites (TTCnnnGAA, perfect match), up to 10% of these are occupied by STATs at any given moment. However, the probability of being bound by a STAT protein is not equal for all GAS sites. John et al. demonstrated that up to 95% of *de novo* glucocorticoid receptor binding sites are pre-determined by chromatin accessibility [48]. In accordance with this, our meta-analysis of genome-wide STAT binding sites in 29 different cell contexts showed that the binding of STATs to GAS sites was mostly defined by the cell type compared to other features such as the type of STATs and the cytokine (Figure 1B). Therefore, the capacity of STATs to access specific GAS sites seems to be pre-determined by the cell type via open chromatin and this notion was validated by previous studies using DNase-seq and STAT5 ChIP-seq in mouse liver [16,49]. In support of this, each cell type displayed a unique STAT binding pattern. The majority of the STAT-bound GAS sites were located near genes with cell-specific expression patterns. This result can explain the cell-specific aspect of STATs that transmits signals for the growth-, survival- and differentiation-related genes corresponding to a given cell type. In contrast to cell-type restricted binding obtained for most sites, we detected 116 highly conserved GAS sites whose recognition by STATs transcended cell types. These CRCCs, which were targeted by any member of the STAT family regardless of cell type and cytokine stimulus, included classical JAK-STAT signature genes, such as *Stat1*, *Socs2*, *Socs3*, *Cish*, *Ifnar2* and *Irf9* genes. Thus, our analysis strengthens previous knowledge that STATs recognize GAS motifs nearby target genes upon cytokine induction and further shows that STATs target cell-type specific as well as common JAK-STAT signature genes.

A previous study using an aneuploid mouse strain carrying human chromosome 21 revealed that transcriptional outputs are determined primarily by genetic sequence besides epigenetic and cellular environment [50]. Several studies demonstrated that nucleosome positions are also determined by nucleotide sequences and therefore, successfully predicted ~50% of *in vivo* nucleosome positions solely based on DNA sequences [51,52]. These findings highlighted the importance of DNA sequences involved in gene regulation. In this regard, binding motifs for distinct transcription factors were enriched around the center of STAT binding sites in specific cell types, suggesting that cell-specific gene regulation of STATs might be driven by cooperative activity of cell-type restricted or enriched TFs. For instance, STAT5 is known to interact with RUNX1 physically *in vitro* which is corroborated by our finding in T cells [39]. The binding motif of HNF4A, which is a key TF in liver [53], coincided significantly with STAT5 binding sites in liver, while none of the other STATs showed any significant association with HNF4A in the different cell types. CEBPA, which is sufficient to promote differentiation of growth-arrested 3T3-L1 cells [54], was significantly over-represented within flanking regions of STAT5 in 3T3-L1 cells. Indeed, the integration of nine TF ChIP-seq data sets from liver and 3T3-L1 cells revealed that STAT5 coincided with cell-enriched TFs. CEBPA, CEBPB, CEBPD and GR coincided with STAT5 in the 3T3-L1 cells, while CEBPA, FOXA1, FOXA2 and HNF4A significantly associated with STAT5 binding sites in female mouse liver. However, E2F4 and p300 were not significantly associated with the STAT5 binding sites in both cell types suggesting that only defined cell-specific co-TFs are related with STATs. Moreover, this finding is supported by a recent study demonstrating that SMAD3, a master transcription factor generating cell-type specific effects of TGFβ signaling, coincided with OCT4 in ES cells, MYOD1 in myotubes and PU.1 in pro-B cells [55]. Collectively, the cooperative activity of STATs with associated TFs appeared to control cell-type specific genes in concordance with previous studies [9,55], while the accessibility of their target GAS sites seems to be pre-determined by epigenetic features including chromatin configurations [48]. Future studies will be required to elucidate which TFs are pioneer factors that recruit co-TFs and/or influence chromatin modifications or bystanders.

## Methods

### ChIP-seq data sets

All data were downloaded from the GEO website (http://www.ncbi.nlm.nih.gov/geo/) [8]. A list of all ChIP-seq data sets can be found in Additional file 2. If aligned files were not provided, we downloaded corresponding unaligned files (.fastq) from the SRA website (http://www.ncbi.nlm.nih.gov/sra) and mapped sequenced reads (tags) to the mouse reference genome (mm9) using the Bowtie aligner with the same parameters as described previously [56,57]. All data sets were converted to BED files (mm9) (http://genome.ucsc.edu/FAQ/FAQformat).

### Data processing

Systematic evaluations of available peak-calling algorithms demonstrated that there are substantial variations in sensitivity and specificity among the programs [20-22]. To identify significant peaks representing STAT binding sites, we analyzed the BED files with three independent peak-calling programs as suggested by Chen et al. [20]: MACS (version 1.4.2), HOMER (version 3.10) and Qeseq (version 0.2.2) with default parameters. Next, all the identified peaks were merged into a single data set. All the merged peaks were categorized into three classes (high-, intermediate- and low-confidence) according to the number of algorithms that detected the peaks (Additional file 1). The low-confidence peaks seem to be false positives since only one of the algorithms detected the regions as binding sites (Figure 1A). Therefore, we only used high- and intermediate-confidence peaks for the rest of the analyses.

### Unsupervised clustering

To estimate overall similarity of genomic STAT binding sites, the mouse genome was divided into 500-bp bins and the numbers of overlaps were calculated between all possible pairs. Hierarchical clustering was performed using the Cluster 3.0 program [58] with the average linkage algorithm. The percentage of the overlaps was used to draw the heat map in Figure 1B.

### Motif analysis

PeakSplitter was used to pinpoint the centers of STAT binding sites with corresponding wig files generated by MACS [59]. To identify significantly over-represented motifs around the centers of STAT binding sites, a web-based *de novo* motif identification program called MEME-ChIP was used with the default setting (http://meme.sdsc.edu/meme/) [35]. The MEME-ChIP program predicts the top three motifs by E-value, which is an estimate of the expected number of motifs in a similarly sized set of random sequences. The top three significant motifs in each set of the top 600 STAT binding sites (+/− 75 bp around the peak centers, sorted by the height of peaks) are shown in Figure 4A. In order to verify the identified motifs, TOMTOM, which compares identified motifs with the known motifs [36], was used.

### Co-transcription factor identification

To identify co-transcription factors in Figure 4B, we used a custom Perl script (MOODS algorithm) with available 130 TFBS position frequency matrices (p value < 0.001, http://jaspar.cgb.ki.se/) [26]. The script is available as Additional file 6. For each TFBS matrix, the number of STAT binding sites containing at least one TFBS within 150 bp (−75 bp ~ peak center ~ +75 bp) was counted and defined as motif-covered sites. The proportion of the motif-covered sites was calculated as ratio of motif-covered sites to total number of sites. The motif enrichment score was calculated using the MOODS algorithm implemented in the script. For each site, the highest motif enrichment score for each TFBS was used. The motif-covered sites and motif enrichment score were then normalized with the values from the same calculation of a background set containing 100,000 random regions (150 bp). To get significantly associated co-TFs with a given set of STAT binding sites, we set the normalized motif-covered site threshold as 0.2 and the motif enrichment score threshold as 1.5. The TFBSs above the thresholds were regarded as significantly associated co-TFs with a given STAT.

### Estimation of empirical P-values

To estimate the significance of STAT5 binding (−10 kb ~ peak center ~ + 10 kb) to gene expression (Figure 5B), we randomly resampled genes among all genes with replacement (the size of the resample was equal to the size of the given STAT5 target genes) and the mean expression values of the resampled set were calculated. This procedure was repeated 10000 times. Then, P-values were empirically computed as the number of times the mean value of a randomly-resampled set was greater than or equal to the observed mean expression value.

### Data access

All data used in this study was downloaded from the GEO web site (http://www.ncbi.nlm.nih.gov/geo/) and detailed information can be found in Additional file 2.

## Abbreviations

CRMs: *Cis*-regulatory modules; CSCC: Common STAT controlled CRMs; GAS: Gamma interferon-activated sequence; ChIP-seq: Chromatin immunoprecipitation followed by high throughput sequencing; RNA-seq: RNA-sequencing; TFs: Transcription factors; TSS: Transcription start site; TFBS: Transcription factor binding site; MEF: Mouse embryonic fibroblast; ES cell: Embryonic stem cell.

## Competing interests

The authors declare that they have no competing interests.

## Authors’ contributions

Experimental design (KK, GWR and LH), data analysis (KK), discussion (KK, GWR and LH), manuscript preparation (KK, GWR and LH). All authors read and approved the final version of manuscript.

## Supplementary Material

Additional file 1 **Peak-calling analysis pipeline used in this study.** A figure showing the peak-calling analysis pipeline. Click here for file

Additional file 2 **Summary of processed ChIP-seq data sets.** A table summarizing processed ChIP-seq data sets. Click here for file

Additional file 3 **Functional annotations of cell-specific STAT binding sites.** A figure showing the functional annotations of cell-specific STAT binding sites. Click here for file

Additional file 4 **Genes near the CSCCs.** A table of genes near the CSCCs. Click here for file

Additional file 5 **Motif prediction with STAT4 and STAT6 binding sites.** De novo motif prediction with top 600 binding sites of STAT4 and STAT6. Click here for file

Additional file 6 **Custom perl script predicting co-transcription factors.** A custom perl script using the MOODS algorithm. Click here for file
