# Supplementary material for: Comprehensive meta-analysis of Signal Transducers and Activators of Transcription (STAT) genomic binding patterns discerns cell-specific cis-regulatory modules
Source: BMC Genomics. 2013 Jan 16;14:4. doi: 10.1186/1471-2164-14-4 (PMC3564941; doi:10.1186/1471-2164-14-4)
Supplement: Additional file 1 — Peak-calling analysis pipeline used in this study. A figure showing the peak-calling analysis pipeline. [file 1471-2164-14-4-S1.pdf]

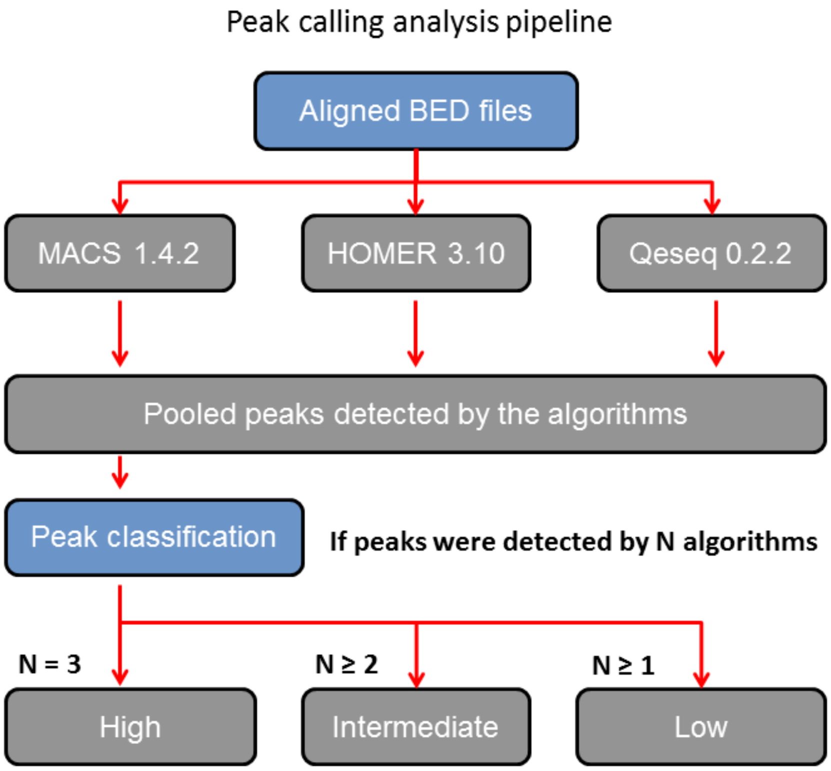

Additional file 1. Peak-calling analysis pipeline used in this study. To achieve robustness of peak detection, we used three peak-calling algorithms called MACS, HOMER and Qeseq. This approach classified peaks into three categories (high, intermediate and low). The high-confidence peaks were defined as the peaks which were detected by all three algorithms, whereas the intermediate- or low-confidence peaks were the peaks identified by either any two or one program(s). This pipeline is to become an essential step since various peak-calling programs have the pros and cons of detecting peaks towards different data sets.
