# Supplementary material for: Comprehensive meta-analysis of Signal Transducers and Activators of Transcription (STAT) genomic binding patterns discerns cell-specific cis-regulatory modules
Source: BMC Genomics. 2013 Jan 16;14:4. doi: 10.1186/1471-2164-14-4 (PMC3564941; doi:10.1186/1471-2164-14-4)
Supplement: Additional file 3 — Functional annotations of cell-specific STAT binding sites. A figure showing the functional annotations of cell-specific STAT binding sites. [file 1471-2164-14-4-S3.pdf]

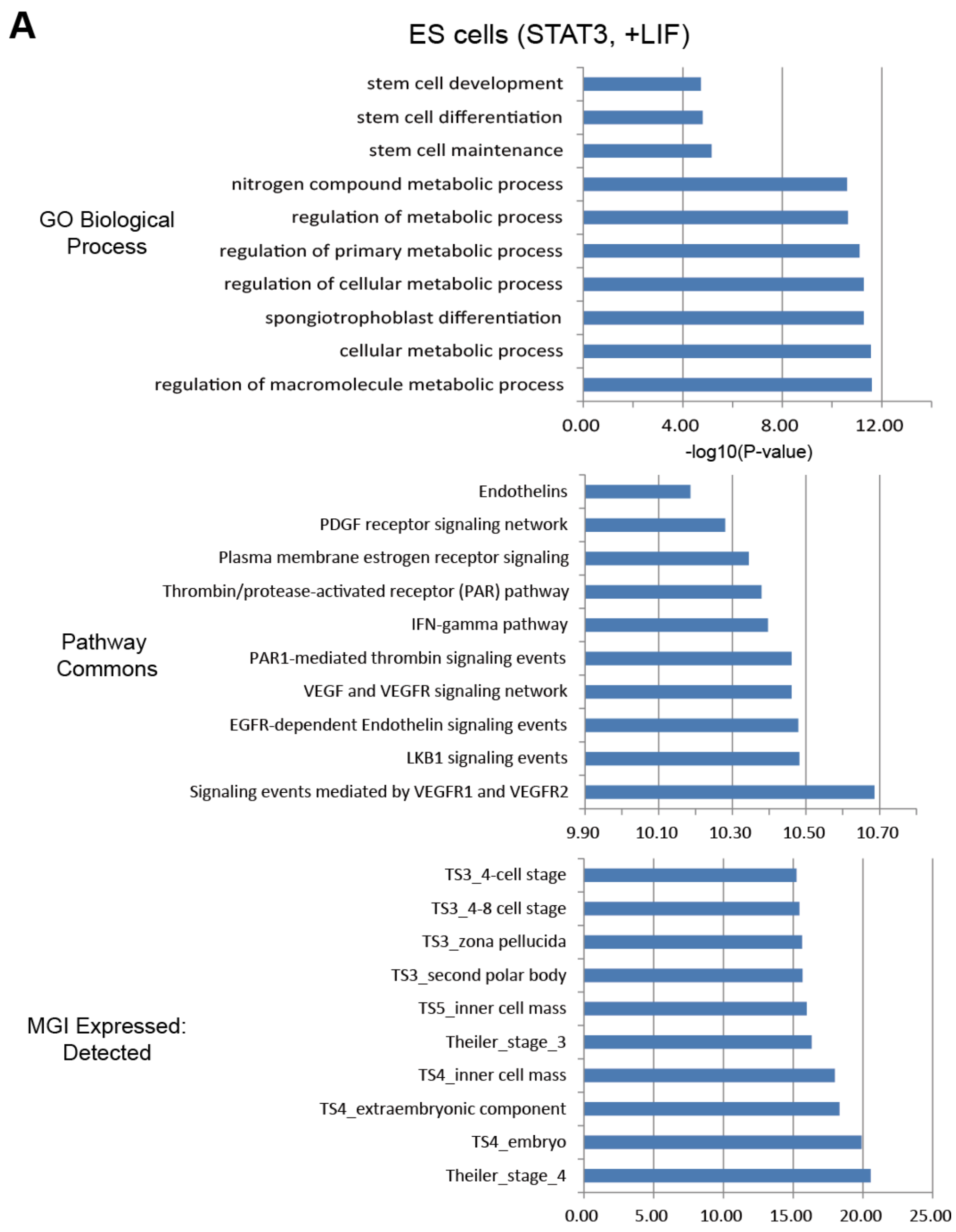

Additional Figure 3. Functional annotations of cell-specific STAT binding sites. The GREAT program assigned biological meaning to the identified STAT binding sites using the known or predicted function of genes based on available gene ontology databases. Significantly associated functions of genes around top 1000 STAT binding sites (sorted by peak height) were inferred. (**A**) STAT3 binding sites in ES cells with LIF. (**B**) STAT5 binding sites in female liver with GH. (**C**) STAT5 binding sites in T cells with IL-2. (**D**) Overexpressed STAT5 binding sites in Stat5$^{-/-}$ MEFs with GH. (**E**) STAT5 binding sites in 3T3-L1 cells with adipogenic inducers. (**F**) STAT1 binding sites in macrophage with IFNγ. P-value - binomial bonferroni P-value.

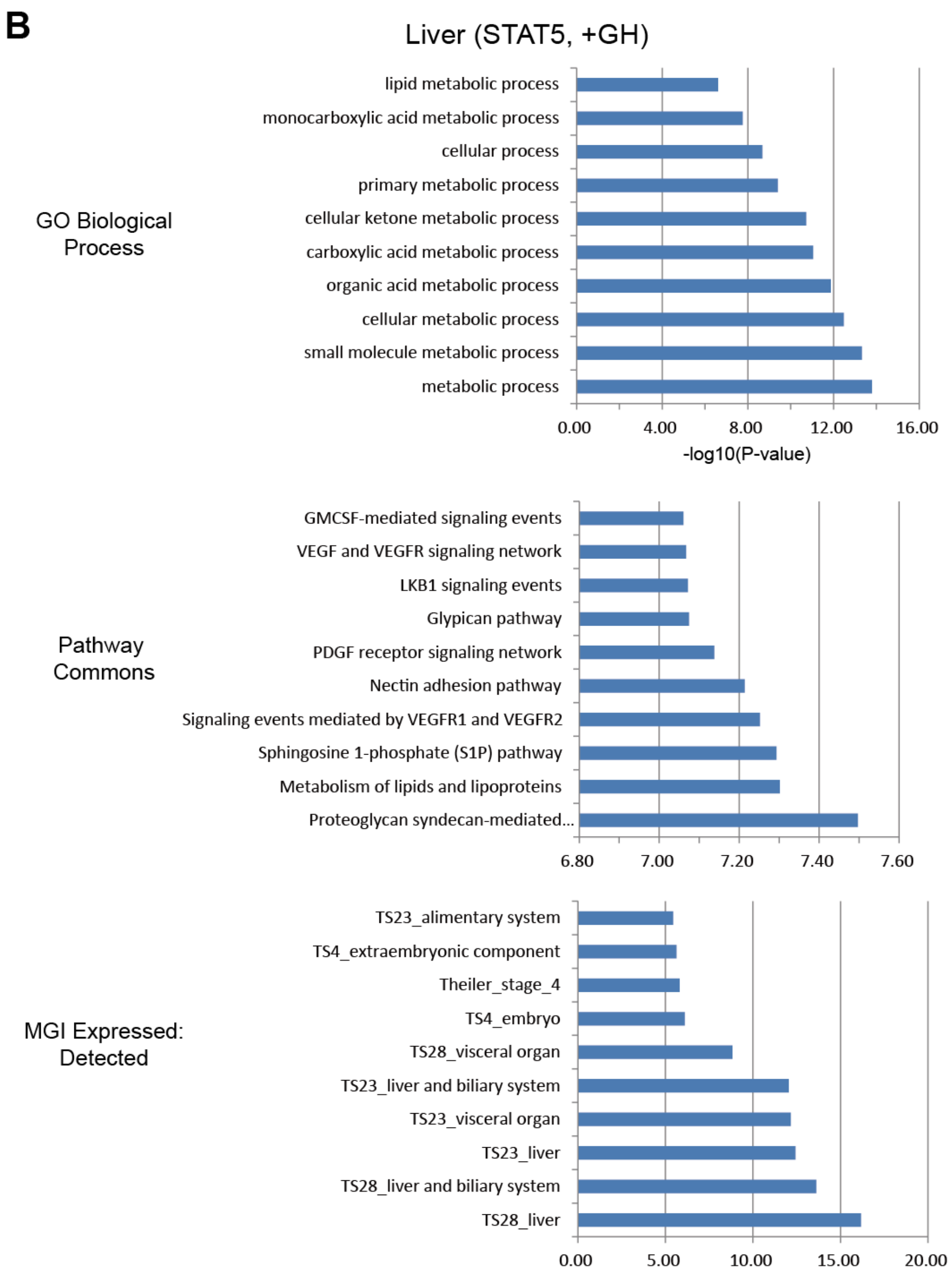

Additional Figure 3. Functional annotations of cell-specific STAT binding sites (continued).

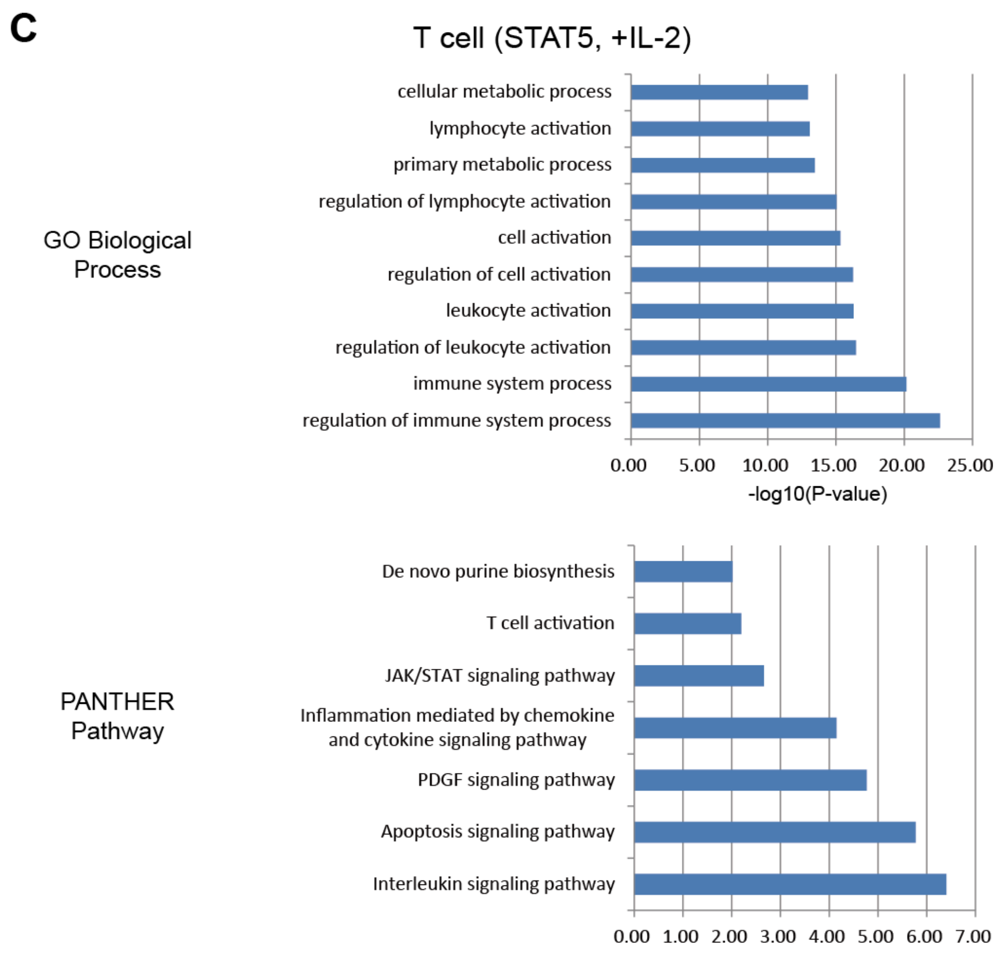

Additional Figure 3. Functional annotations of cell-specific STAT binding sites (continued).

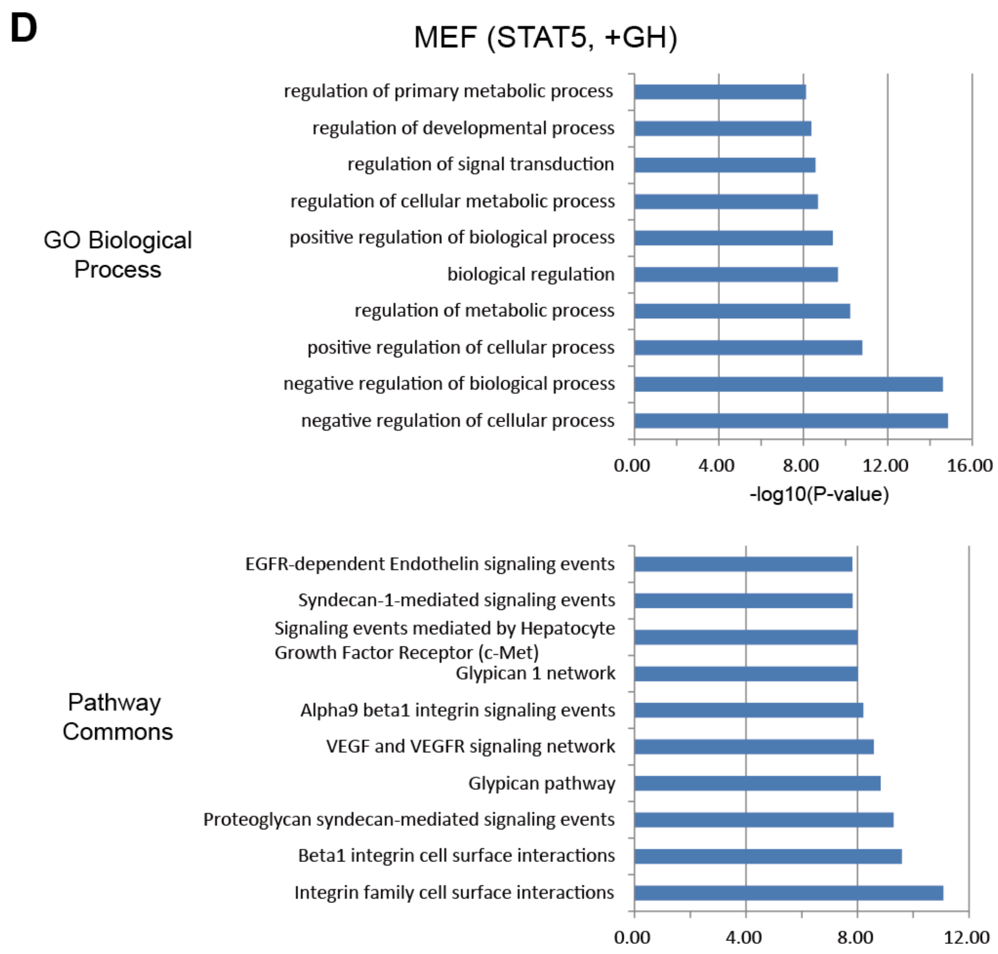

Additional Figure 3. Functional annotations of cell-specific STAT binding sites (continued).

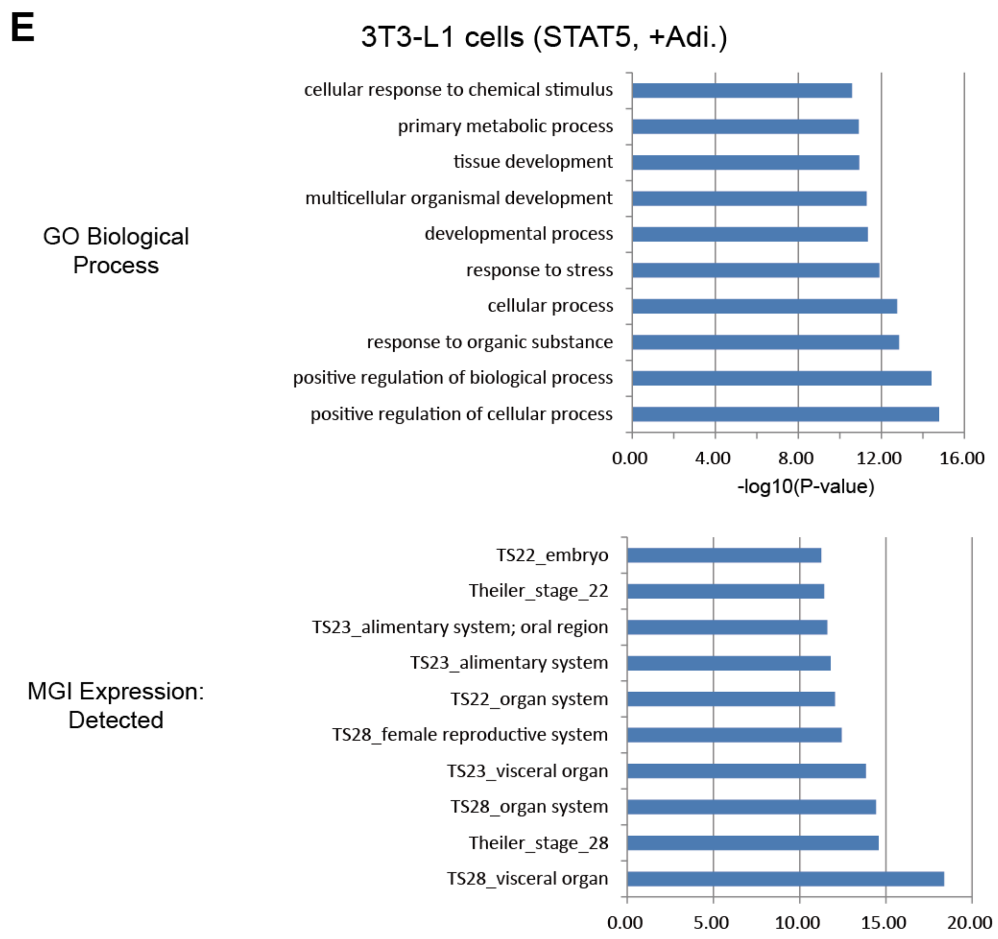

Additional Figure 3. Functional annotations of cell-specific STAT binding sites (continued).

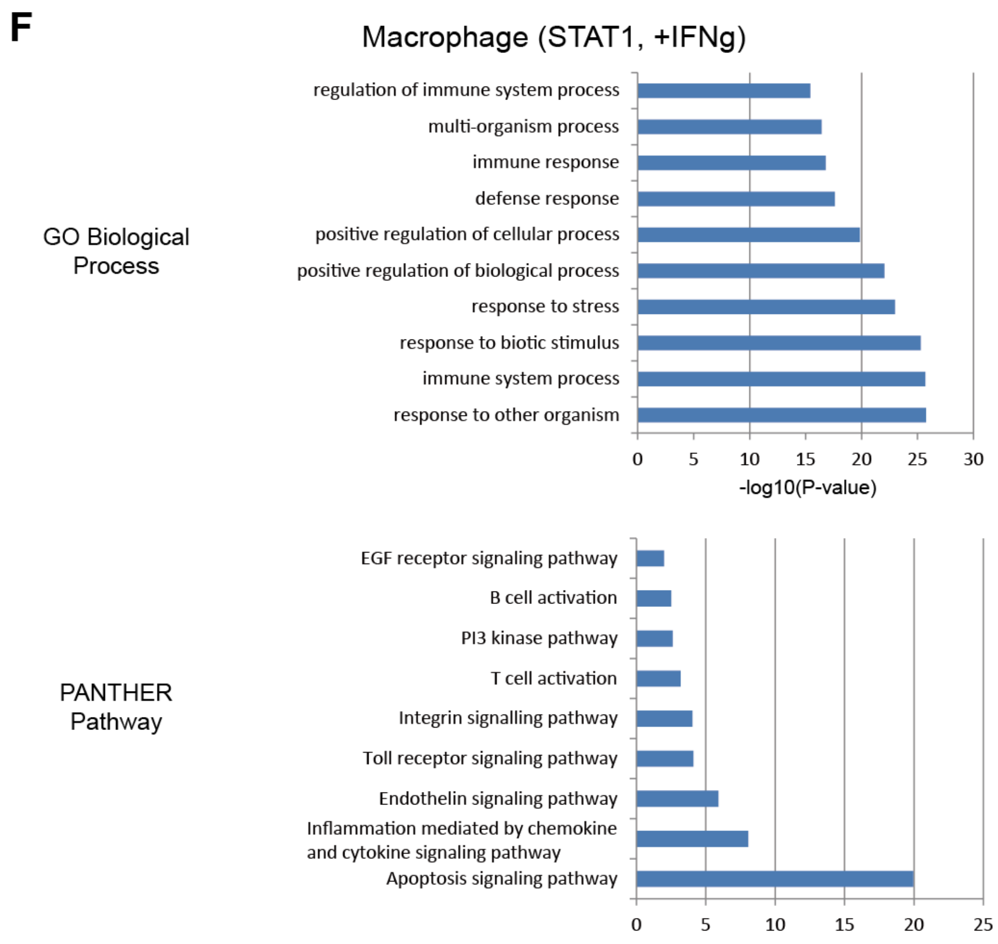

Additional Figure 3. Functional annotations of cell-specific STAT binding sites (continued).
