# Supplementary material for: Comprehensive meta-analysis of Signal Transducers and Activators of Transcription (STAT) genomic binding patterns discerns cell-specific cis-regulatory modules
Source: BMC Genomics. 2013 Jan 16;14:4. doi: 10.1186/1471-2164-14-4 (PMC3564941; doi:10.1186/1471-2164-14-4)
Supplement: Additional file 5 — Motif prediction with STAT4 and STAT6 binding sites. De novo motif prediction with top 600 binding sites of STAT4 and STAT6. [file 1471-2164-14-4-S5.pdf]

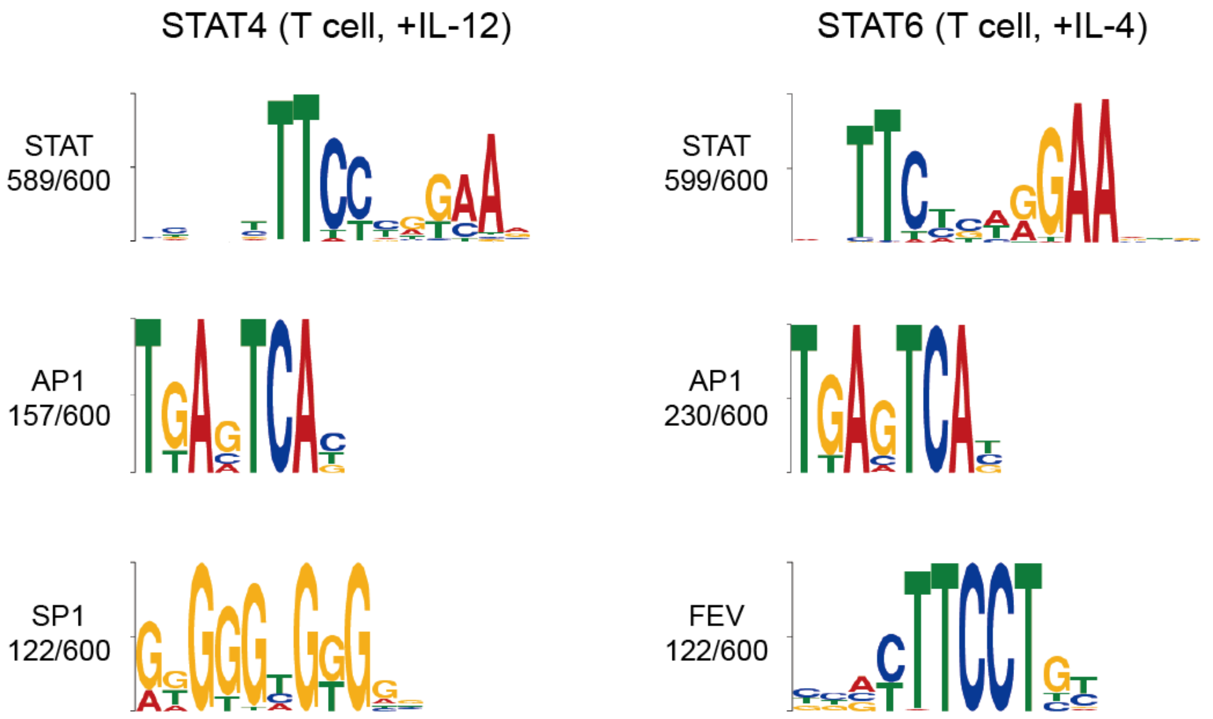

Additional file 5. Predicted motifs in top 600 STAT4 and STAT6 binding sites. The same motif analysis (Fig. 4A) was performed with top 600 (ranked by peak height) STAT4 and STAT6 binding sites. The number of sites containing the predicted motifs is shown.
